# Facial image inpainting for big data using an effective attention mechanism and a convolutional neural network

**DOI:** 10.3389/fnbot.2022.1111621

**Published:** 2023-01-12

**Authors:** Xiaoman Lu, Ran Lu, Wenhao Zhao, Erbin Ma

**Affiliations:** Department of Mathematics, College of Science, Northeastern University, Shenyang, Liaoning, China

**Keywords:** big data artificial intelligence (AI), deep learning algorithm, deep learning-based facial image inpainting, generative adversarial network, convolutional neural networks

## Abstract

Big data facial image is an important identity information for people. However, facial image inpainting using existing deep learning methods has some problems such as insufficient feature mining and incomplete semantic expression, leading to output image artifacts or fuzzy textures. Therefore, it is of practical significance to study how to effectively restore an incomplete facial image. In this study, we proposed a facial image inpainting method using a multistage generative adversarial network (GAN) and the global attention mechanism (GAM). For the overall network structure, we used the GAN as the main body, then we established skip connections to optimize the network structure, and used the encoder–decoder structure to better capture the semantic information of the missing part of a facial image. A local refinement network has been proposed to enhance the local restoration effect and to weaken the influence of unsatisfactory results. Moreover, GAM is added to the network to magnify the interactive features of the global dimension while reducing information dispersion, which is more suitable for restoring human facial information. Comparative experiments on CelebA and CelebA-HQ big datasets show that the proposed method generates realistic inpainting results in both regular and irregular masks and achieves peak signal-to-noise ratio (PSNR) and structural similarity (SSIM), as well as other evaluation indicators that illustrate the performance and efficiency of the proposed model.

## 1. Introduction

With the rapid development of computer vision technology, digital images (Singh and Goel, 2020) have become the mainstream of facial image acquisition. Normally, people usually rely on electronic devices to obtain facial images; however, watermark occlusion, smear, part of the area missing, and other problems often appear in the transmission process of digital images (Baeza et al., 2009), preservation (Meyers and Scott, 1994), and post-processing (Shen and Kuo, 1997), damaging the quality of the facial image and resulting in poor visual feeling (Parmar, 2011). To solve the abovementioned problems, related scholars began to study these kinds of problems and proposed a series of novel inpainting approaches.

Image inpainting is a very challenging task in image processing (Elharrouss et al., 2020), and its purpose is to restore and complete the missing or defaced image part. A new image needs to be inferred and constructed according to the contextual information of the damaged image and the overall image structure (Jin et al., 2021). The restored image should have clear textures and natural boundary pixels and conform to human visual perception. Compared to other image inpainting tasks, some similar image blocks cannot be found in other facial image areas (Yang et al., 2020). For example, it is difficult to infer a reasonable nose image based on the surrounding areas when the nose part is missing, which may lead to an imbalance proportion of facial images. For this problem, it is necessary to reconstruct images that satisfy human visual perception according to a large amount of prior information and contextual semantics (Yeh et al., 2017).

Before deep learning methods were proposed, there were two kinds of theoretical research in image inpainting, including partial differential equations- and texture-based methods. Bertalmio et al. (2000) used partial differential equations to diffuse neighborhood pixel information to the missing area using an isograd direction field. For images with small missing areas, satisfactory results can be achieved. However, it is not ideal for images with large missing areas because this method does not consider the semantic information of the image context. Efros and Leung (1999) first proposed the generation of patch blocks with similar textures using the extracted texture information of the missing regions and then the use of the generated patch blocks to fill in the missing regions. The disadvantage of this method is that, although the missing area is filled, the filled area is compact overall from the content level but not from the pixel level. In other words, the repair result is not smooth enough, with many traces of artificial processing. Criminisi et al. (2004) established a block image restoration method based on texture synthesis. In this method, a pixel randomly selected on the image's missing area boundary is taken as the center to choose a certain size image texture block, which is then used to repair the missing area. This image inpainting method can fill in more appropriate texture information for the missing areas, but because the contextual semantic information of an image is ignored and contextual semantics of the repaired image becomes incoherent, the complex facial image inpainting task cannot be completed.

Deep-learning-based facial image inpainting technology (Qin et al., 2021) is more suitable for a variety of restoration scenarios than traditional image restoration methods. The feature distribution dataset learned by a neural network is more suitable for facial image restoration with a large missing area and random damage. Not only are the texture details accurate but also are the contours harmonized, and the facial image conforms to the contextual semantics (Wei et al., 2019). After ongoing in-depth research by relevant scholars, deep learning-based image repair methods have produced a number of results.

Pathak et al. (2016) used a context encoder to complete an image repair task, which was the first image inpainting method based on a generative adversarial network (GAN). The generator is divided into an encoder and a decoder (Sun et al., 2018). The encoder is responsible for compressing and extracting feature information from an incomplete image, and the decoder is responsible for restoring an input-compressed feature to the image. In this method, the context encoder can achieve a good repair effect, but the generation antagonism losses adopted by the context encoder considers only local information of an incomplete region and not the overall semantic coherence of the image. Iizuka et al. (2017) adopted a global–local double discriminator to improve the context encoder. A local discriminator was applied to the repair result of an incomplete area, and a global discriminator was applied to the overall repair result. This design ensured not only the accuracy of the repair area but also the integrity of the final result. However, the prediction results of this method are still inaccurate when the large area facial image is missing. Yang et al. (2017) proposed the use of content and texture generation networks to complete the image repair task. The content generation network is responsible for inferring the semantics and global structure of an image, while the texture generation network is responsible for generating high-frequency details of an image. Compared to previous methods, this method solves the problem of high-resolution image repair. Yan et al. (2018) added a shift-connection layer on the basis of the U-Net network. In this method, pixel information from known regions is transferred to the corresponding missing regions to assist the image repair generated in the process of guided loss minimization, which encodes and decodes the distance between the distribution and the true distribution. However, due to the shortcomings of a simple structure of the algorithm, it is not effective in restoring facial images, which have problems such as blurred edges.

Although GANs are widely used in the field of image inpainting, they still rely too much on the self-generation ability of generative networks and have many problems to solve. For example, when the texture structure of a facial image is more complex, it is easy to appear fuzzy, semantic incoherence and other phenomena. When the local feature of the facial image is not clear, the information stored in the model is too large and network training is prone to information overload.

To solve these problems, based on the normalization of the feature layer output in the GAN and the guiding role of the attention mechanism in image detail inpainting, this study proposes a facial image inpainting method using a multistage GAN based on a global attention mechanism (GAM) named CLGN, where a generative network can accelerate the training speed and improve training stability through feature layer output normalization. By using step coiling instead of up-sampling and full-connection layers, convolution can play a good role in extracting image features. Meanwhile, GAM (Liu et al., 2021) was introduced to enhance the guiding role of important features during the image inpainting process. In addition, a U-Net skip-connection (Ronneberger et al., 2015) was introduced between the encoder and the decoder to reduce information loss due to down-sampling and to optimize texture consistency. The loss function is used as an important factor to measure the generated image quality and loss (Gao and Fang, 2011), weighted reconstruction loss, perceptual loss, style loss, and total variation (TV) loss, which were combined to optimize the total loss of the generated network for model training.

Our study provides the following contributions:

- Building a multistage (crude-local-global) generative network CLGN to capture feature information from receptive fields of different sizes and enhance presentation capabilities.
- Adding GAM to magnify the interactive features of the global dimension while reducing information dispersion, which is more suitable for restoring human facial information.
- The proposed CLGN produces photorealistic and plausible inpainting results on two datasets, CelebA and CelebA-HQ. The remainder of this paper is organized as follows: Section 2 introduces the relevant theories used in our proposed method. Section 3 shows the observation and motivation, the network architecture, and loss functions. Section 4 focuses on comparative and ablation experiments of our methods. Section 5 concludes and discusses future research.

## 2. Related theory

### 2.1. Generative adversarial networks

**Figure 1:**
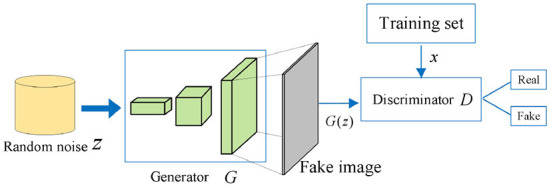
The model of generative adversarial networks (GANs).

A generative adversarial network was proposed by Goodfellow et al. (2014). In recent years, GANs have been extensively studied in combination with other machine learning algorithms in some specific applications, such as semi-supervised learning (Odena, 2016), transfer learning (Cho et al., 2017), and reinforcement learning (Wang et al., 2020), and are widely used in image inpainting. GAN has made a considerable breakthrough in image inpainting by producing realistic images. The core idea of GAN comes the “two-player zero-sum game” in game theory (Ge et al., 2018), in which networks are optimized by cheating each other between generators and discriminators, resulting in a Nash equilibrium. The GAN consists of a generative network *G* and a discriminant network *D*, and its structure is shown in Figure 1.

By learning the probability distribution mapping *P*_*data*_ of the real data, the generative network *G* is expected to output content *G*(*z*) close to the real data. The discriminant network *D* needs to identify the source of the input data as much as possible, i.e., classify *x* and *G*(*z*). When the discriminant network *D* cannot distinguish data sources, network performance is optimal. Its objective function is as follows:


$$\begin{array}{l} \underset{G}{\min}\underset{D}{\max}V\left(D,G\right)=E_{x\sim P_{data}}\left[\log\left(D\left(x\right)\right)\right]\\ \;+E_{z\sim P_{g}}\left[\log\left(1-D\left(G\left(z\right)\right)\right)\right], \end{array}$$

where *G* represents a generative network, *D* represents a discriminant network, *E*(•) represents the mathematical expectation, *V* represents the objective function, *x* represents the sample, *z* represents random noise, and *P*_*data*_ represents the distribution of the real sample.

### 2.2. Visual geometry group network

The visual geometry group network (VGGNet) was proposed by Karen Simonyan and Andrew Zisserman of the Visual Geometry Group at the University of Oxford (Simonyan and Zisserman, 2015). An outstanding contribution of VGGNet is to demonstrate that small convolutions can effectively improve performance by increasing network depth. VGG expertly inherits the mantle of Alexnet while also exhibiting the characteristics of a deeper network layer.

**Figure 2:**
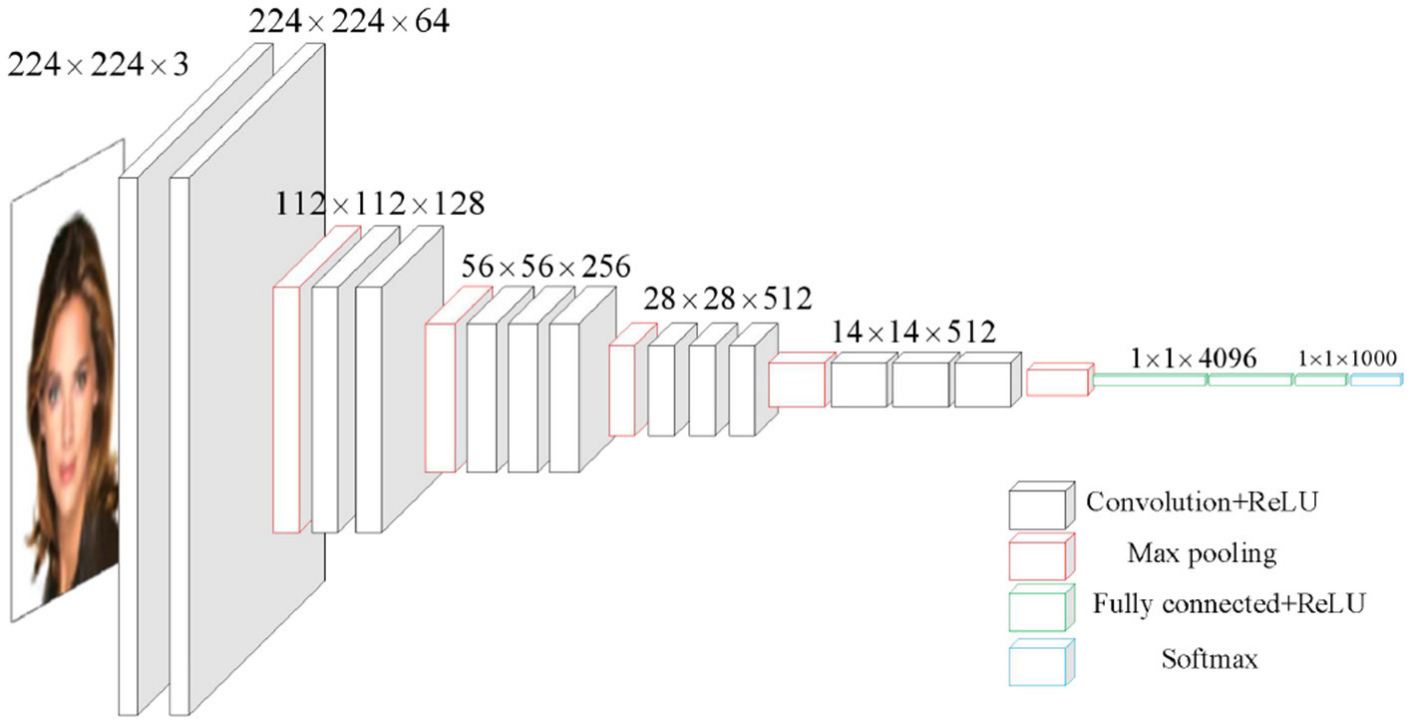
The structure of a VGG16 module. The face images are adapted from the celeba-HQ dataset, which comes from Karras et al. (2017).

The structure of VGGNet is shown in Figure 2 (Noh et al., 2015) and consists of five convolutional layers, three fully connected layers, and softmax output layers. These layers are separated by max-pooling (maximization pool), and the activation units of all hidden layers adopt the ReLU function. VGG uses multiple convolution layers with smaller convolution kernels (3 × 3) to replace one convolution layer with a larger convolution kernel. On the one hand, parameters can be reduced. On the other hand, it is equivalent to perform more non-linear mapping, which can increase the network's ability to fit and express.

### 2.3. Global attention mechanism

In recent years, attention mechanisms have been widely used in many applications (Zn et al., 2021). The convolutional block attention module (CBAM) (Woo et al., 2018) sequentially places the channel and spatial attention operation, while the bottleneck attention module (BAM) (Park et al., 2018) does it in parallel. However, both of them ignore channel-spatial interactions and consequently lose cross-dimensional information.

Therefore, GAM that boosts network performance by keeping the amount of information to a minimum and zooming in on the global interaction representation has been proposed. GAM (Liu et al., 2021) is a simple yet effective attention module that reserves information to magnify the “global” cross-dimensional interactions. The GAM adopts the sequential channel-spatial attention mechanism from CBAM (Woo et al., 2018), which is an elementary yet practical attention module for feed-forward convolutional neural networks. CBAM can be regarded as a dynamic selection process for inputting important information into an image, which significantly improves the performance level of many computer vision tasks and plays an important part in image inpainting with complex image structures.

**Figure 3:**
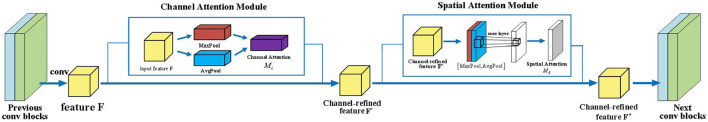
The model of convolutional block attention module (CBAM).

The internal structure of CBAM is shown in Figure 3. We set the intermediate feature map *F* ∈ ℝ^*C* × *H* × *W*^ as input. CBAM deduces an attention map in two separate dimensions, channel and space, which are shown as a one-dimensional (1D) channel attention map $M_{c}\in {\mathbb{R}}^{C\times 1\times 1}$ and a two-dimensional (2D) spatial attention map $M_{s}\in {\mathbb{R}}^{1\times H\times W}$. In conclusion, the general process of the attention module can be represented as:


$$\left\{ \begin{array}{l} \;F^{\prime }={\text{M}}_{\text{c}}(F)⊗F,\\ \;F^{\prime \prime }={\text{M}}_{s}(F^{\prime })⊗F^{\prime }, \end{array}\right.$$

where ⊗ indicates an element-wise multiplication and *F*″ is named as the final refined output. The detailed operations for each module are described as follows.

For the channel attention module, first, we applied both average pool and max pool operations to gather spatial information, producing two disparate spatial context descriptors: ${\text{F}}_{avg}^{c}$ and ${\text{F}}_{max}^{c}$ representing the max-pooling features. Then, we sent two descriptors to a shared network to produce a channel attention map $M_{c}\in {\mathbb{R}}^{C\times 1\times 1}$.

To cut down parameter overhead, we set the hidden activation size ℝ^*C*/*r* × 1 × 1^, where *r* is the reduction ratio. Finally, the output feature vectors are conflated by element-wise summation after we applied the shared network to each descriptor. In conclusion, the channel attention is represented by:


$$\begin{array}{l} \begin{array}{l} {\text{M}}_{c}\left(\text{F}\right)=\sigma \left(MLP\left(AvgPool\left(\text{F}\right)\right)+MLP\left(MaxPool\left(\text{F}\right)\right)\right)\\ \;=\sigma \left({\text{W}}_{1}\left({\text{W}}_{0}\left({\text{F}}_{avg}^{c}\right)\right)+{\text{W}}_{1}\left({\text{W}}_{0}\left({\text{F}}_{\max}^{c}\right)\right)\right), \end{array} \end{array}$$

where σ indicates the sigmoid function, $W_{0}\in {\mathbb{R}}^{C/r\times C}$, and $W_{1}\in {\mathbb{R}}^{C\times C/r}$.

For the spatial attention module, firstly, we introduce the average and maximum pools on the canal axis and connect them to establish an adequate feature description so that spatial attention can be calculated. In cascaded feature descriptors, we used the convolution layers to create a spatial attention pattern $M_{s}\left(F\right)\in R^{H\times W}$ in which positions of emphasis or suppression can be encoded. In particular, we created two 2D maps: $F_{avg}^{s}\in {\mathbb{R}}^{1\times H\times W}$ and $F_{max}^{s}\in {\mathbb{R}}^{1\times H\times W}$, which reflect the average characteristics of swimming pools in the canal and their maximum characteristics. Then, a 2D spatial attention table is output after being connected to the standard convolution layer and convoluted at its end. Spatial attention is calculated as follows:


$$\begin{array}{l} \begin{array}{l} {\text{M}}_{s}\left(F\right)=\sigma \left(f^{7\times 7}\left(\left[AvgPool\left(\text{F}\right);MaxPool\left(\text{F}\right)\right]\right)\right)\\ \;=\sigma \left(f^{7\times 7}\left(\left[{\text{F}}_{avg}^{s};{\text{F}}_{\max}^{s}\right]\right)\right), \end{array} \end{array}$$

where σ represents the sigmoid function and *f*^7 × 7^ denotes a convolution operation with the filter size of 7 × 7.

## 3. Proposed method

### 3.1. Observation and motivation

Traditional image inpainting methods are based on texture extension (Bertalmio et al., 2000) or similar block matching (Criminisi et al., 2004). These methods do not repair some damaged images with large missing areas and complex structures of missing areas. Especially, in facial image inpainting, the big challenge is how to ensure the overall consistency of the inpainting results and restore the missing details and textural features.

In this study, we put forward a facial image inpainting method using an attention-based multistage GAN followed by a crude-local-global framework. Considering that missing areas of different sizes can be solved, the proposed network contains a three-stage network for image inpainting to combine the networks with different receptive fields. The network structure and the corresponding loss functions are described in Section 3.2.

### 3.2. Network architecture

#### 3.2.1. Crude inpainting network

Our crude inpainting network *Net*_*C*_ employs an encoder–decoder framework with the addition of a skip connection, consisting of eight down- and up-sampling operations. We used long skip connections to transmit information from the encoder to the decoder to restore information lost during down-sampling. The receptive field resolution is 766 × 766 and is nearly three times larger than the input image resolution with a size of 256 × 256.

For a convolutional neural network, a large receptive field is helpful to the whole image inpainting. At the input end, the network receives an input image $I_{in}$ and a binary mask *M*, which describe the missing areas. Note that the missing pixel is equal to 1 and the valid pixel is equal to 0. Meanwhile, at the output end, the network exports an inpainting image $I_{out}^{C}$.

To weaken the blur effect and improve the restoration effect of inpainting images, a patch-based discriminator with spectral normalization was also applied. The inputs for the discriminator were a ground truth image and the inpainting image $I_{out}^{C}$, while the output was a 2D feature map where the shape is ℝ^32 × 32^. The function of the discriminator is to determine whether each element in the feature map is true or false.

#### 3.2.2. Local development network

**Figure 4:**
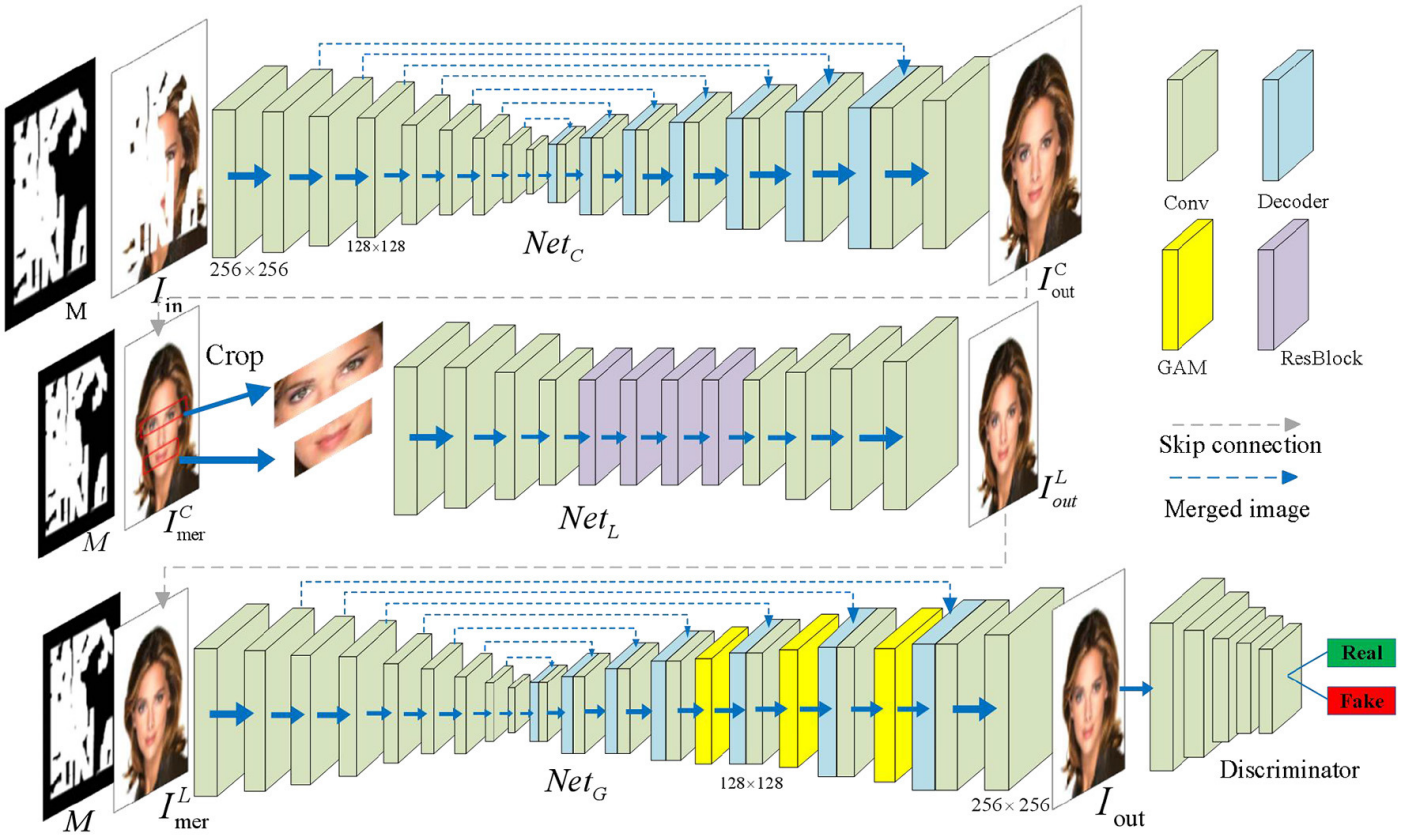
The network architecture of our proposed method CLGN. The purple block in the local development network indicates a two-layer residual block (He et al., 2016). The three yellow blocks represent the GAM attention modules with resolutions of 16 × 16, 32 × 32, and 64 × 64, respectively. The green blocks represent the convolutional layer and the blue blocks represent the decoder. The face images are adapted from the celeba-HQ dataset, which comes from Karras et al. (2017).

To further optimize the local refinement, we designed a surface-deep network called the local refinement network *Net*_*L*_, which includes two down-sampling operations, four residual blocks, and two up-sampling operation, as shown in the middle row of Figure 4.

Due to its surface nature, this network has a small receptive field with the size of 109 × 109 for each output neuron. The local area of the above-mentioned rough inpainting results was then processed in a sliding window manner. Because of this design, some missing areas, such as the local structures and the textures, can be properly repaired by the surrounding local image information. Moreover, this process is not affected by the long distances and content not being filled. In addition, more residual blocks are introduced into this network, which can gradually make the receptive field larger and significantly reduce the model generalization error.

#### 3.2.3. Global attention-based network

After the local refinement network process, some unresolved visual artifacts are properly removed with the help of surrounding local areas. Nevertheless, some missing areas (e.g., facial features such as the eyes or the mouth that are easily mismatched) still need to be better refined when capturing information from the corresponding large surrounding areas. In view of this fact, a global attention-based network is established, which can expand the scope of access to information for a neuron in two ways, i.e., the attention mechanism and a large receptive field.

Considering that a crude inpainting network has enough receptive fields to cover the whole image area, we exploited the basic structure of GAM. Based on this, three CBAMs are added in front of the decoder, aiming to attain a global attention-based network *Net*_*G*_ (see the three yellow blocks in the third row of Figure 4). Moreover, considering that the local development network can already provide relatively correct image restoration results, there is a major trend for a novel network *Net*_*G*_ based on the attention mechanism to become more stable and robust. Some existing studies (Yu et al., 2018, 2019) used the attention mechanisms to calculate the correlation between contextual information and the missing areas. In this study, a lightweight and powerful GAM attention module along two separate dimensions (i.e., channel and spatial) was used. A feature map *F* ∈ ℝ^*C* × *HW*^is given, and the affinity $s_{i,j}\in {\mathbb{R}}^{HW\times HW}$ of *F*_*i*_ and *F*_*j*_ is computed by:


$$\begin{array}{l} s_{i,j}=\frac{exp\left({\hat{s}}_{i,j}\right)}{\sum_{k}^{\;}exp\left({\hat{s}}_{i,j}\right)},{\hat{s}}_{i,j}=<\frac{F_{i}}{\left||F_{i}|\right|},\frac{F_{j}}{\left||F_{j}|\right|}>. \end{array}$$

Note that the weighted average version *F* is $\tilde{F}=F*S\in {\mathbb{R}}^{C\times HW}$ in terms of matrix multiplication.

In the end, we connected *F* and $\tilde{F}$. Then, we introduced a 1 × 1 convolutional layer to maintain the number of inchoative channels *F*.

### 3.3. Loss functions

#### 3.3.1. Reconstruction loss

In terms of pixel-level supervision, we used weighted *l*_1_ loss as the reconstruction loss to measure the distance between the ground truth *I*_*gt*_ and the generated image *I*_*out*_, let:


$$\begin{array}{l} {ℒ}_{valid}{\;}^{\text{C}}=\frac{1}{sum(1_{A}-M)}||(I_{out}^{\text{C}}-I_{gt})⊙(1_{A}-M)|{|}_{1},\\ \;{ℒ}_{hole}{\;}^{\text{C}}=\frac{1}{sum(M)}||(I_{out}^{\text{C}}-I_{gt})⊙M|{|}_{\text{1}}, \end{array}$$

where 1_*A*_ means the indicator function, *I*_*gt*_is the ground truth image, ⊙ is the element-wise product operation, and *sum*(*M*) is the number of non-zero elements in *M*. Then, the pixel-wise reconstruction loss is formulated as:


$$\begin{array}{l} L_{r}^{\text{C}}=L_{valid}^{\text{C}}+\lambda_{h}\cdot L_{hole}^{\text{C}}. \end{array}$$

In addition, the first training target of the local refinement network (*Net*_*L*_) is the weighted reconstruction loss $L_{r}^{L}$, which is the same as Equation (7) except for replacing $I_{out}^{c}$ with $I_{out}^{L}$ in Equation (6).

#### 3.3.2. Adversarial loss

In this study, we used the least square loss function for GAN loss. Least square loss (Mao et al., 2017) not only enhances stability during the training process but also develops generator performance with the aid of more gradients. Then, we define the corresponding loss functions for the crude inpainting network and discriminator as:


$$\begin{array}{l} {\text{I}}_{\text{mer}}^{\text{C}}={\text{I}}_{in}⊙\left(1_{A}-\text{M}\right)+{\text{I}}_{out}^{C}⊙\text{M},\\ L_{\text{G}}^{\text{C}}={E_{\text{I}}}_{\text{mer}}\sim {p_{\text{I}}}_{mer}\left({\text{I}}_{\text{mer}}\right)\left[{\left(D\left(I_{mer}^{C}\right)-1\right)}^{2}\right],\\ L_{D}=\frac{1}{2}E_{\text{I}-pdata\left(I\right)}\left[{\left(D\left(I_{gt}\right)-1\right)}^{2}\right]\\ \;+\frac{1}{2}E_{{\text{I}}_{mer}\sim {p_{\text{I}}}_{mer}\left({\text{I}}_{mer}\right)}\left[{\left(D\left(I_{mer}^{c}\right)\right)}^{2}\right], \end{array}$$

where 1_*A*_ represents the indicator function, *E* means mathematical expectation, $I_{\text{mer}}^{\text{C}}$ is the merged image, and *I*_gt_ is the ground truth image.

#### 3.3.3. Total variation loss

In signal processing, TV denoising is a noise-removal process (Liu et al., 2018). It is based on the principle that signals with excessive and possibly spurious detail have high TV, that is, the integral of the absolute image gradient is high. Following Liu et al. (2018), we used TV loss as a smoothing penalty. The formula is as follows:


$$\begin{array}{l} L_{\text{tv}}^{\text{L}}=||I_{\text{mer}}^{\text{L}}\left(i,j+1\right)-I_{\text{mer}}^{\text{L}}\left(i,j\right)|{|}_{1}\\ \;+||I_{\text{mer}}^{\text{L}}\left(i+1,j\right)-I_{\text{mer}}^{\text{L}}\left(i,j\right)|{|}_{1}. \end{array}$$

where the calculation process is precisely the same as that of $I_{\text{mer}}^{\text{C}}$, i.e., Equation (8).

#### 3.3.4. Perceptual loss

To better renovate the structural and textual information, we apply the perceptual loss (Johnson et al., 2016) based on VGG-16 (Simonyan and Zisserman, 2015), which is trained in ImageNet beforehand. Unlike the pixel-level reconstruction loss and TV loss mentioned above, which are done in pixel space, the perceptual loss is calculated in feature space. Furthermore, perceptual loss is shown by:


$$\begin{array}{l} L_{per}^{\text{L}}=\underset{i}{\sum }\begin{array}{c} ||F_{i}\left(I_{\text{out}}^{\text{C}}\right)-F_{i}\left(I_{\text{gt}}\right)|{|}_{1}\\ +||F_{i}\left(I_{\text{mer}}^{\text{L}}\right)-F_{i}\left(I_{\text{gt}}\right)|{|}_{1} \end{array}, \end{array}$$

where is the feature map of the *i*th layer in the VGG-16 network (Simonyan and Zisserman, 2015), which is pretrained, *i* ∈ {5, 10, 17}.

#### 3.3.5. Style loss

Style loss represents the difference in the Gram matrix between the features of the synthesized image and the features of the style image, ensuring that the style of the generated image matches the style image. Here, we define style loss as follows:


$$\begin{array}{l} L_{sty}^{\text{L}}=\underset{i}{\sum }\begin{array}{c} ||G_{i}\left(I_{\text{out}}^{\text{L}}\right)-{G_{i}}_{i}\left(I_{\text{gt}}\right)|{|}_{1}\\ +||G_{i}\left(I_{\text{mer}}^{\text{L}}\right)-{G_{i}}_{i}\left(I_{\text{gt}}\right)|{|}_{1} \end{array}, \end{array}$$

where $G_{i}\left(\cdot \right)=F_{i}\left(\cdot \right)F_{i}{\left(\cdot \right)}^{T}$ is the Gram matrix.

#### 3.3.6. Style loss

For a crude inpainting network, we summarized the total loss of *Net*_*C*_:$L_{C}=L_{valid}^{C}+\lambda_{h}$.$L_{hole}^{C}+\lambda_{g}\cdot L_{G}^{C}$. It should be noted that we set λ_*h*_ = 6 and λ_*g*_ = 0.1 in all experiments.

For the local development network, the target for the local refinement network *Net*_*L*_ is defined as:


$$\begin{array}{l} L_{L}=L_{valid}^{L}+\lambda_{h}\cdot L_{hole}^{L}+\lambda_{TV}\cdot L_{TV}^{L}+\lambda_{per}\cdot L_{per}^{L}\\ \;+\lambda_{sty}\cdot L_{sty}^{L} \end{array}$$

In our experiments, we discovered that weight losses in Liu et al. (2018) were correspondingly balanced in the order of magnitude, so the weight setting was adopted. We set λ_*h*_ = 6,λ_*tv*_ = 0.1,λ_*per*_ = 0.05, and λ_*sty*_ = 120 in a special way.

For a GAM attention-based global refinement network, we found that the training target G_*G*_ of *Net*_*G*_ is almost consistent with G_*L*_ of *Net*_*L*_, and we only need to replace $I_{out}^{L}$ with $I_{out}^{G}$ in the corresponding positions of G_*L*_.

In this connection, the novel inpainting network CLGN is trained using an “end-to-end” method, and the overall CLGN output becomes the final image inpainting result. The sum total of three subnetworks and a discriminator is the final training loss, i.e., G_*C*_+G_*L*_+G_*G*_+G_*D*_.

## 4. Experiments

### 4.1. Experimental settings

#### 4.1.1. Experimental platform and parameters

For network training, the hardware platform is an AMD EPYC 7302 16-Core Processor CPU, a single GeForce RTX 3090 (31GB), and the software platform is PyTorch1.3.0. During training, each image and mask were resized to 256 × 256 by bicubic interpolation, and there are no data arguments. The Adam optimizer is used with an initial learning rate of 0.0002 for the first 100 epochs and later decays the learning rate to 0 for the next 100 epochs to fine-tune the model. In addition, the first-order momentum was set as β_1_ = 0.5 and the second-order momentum was set as β_2_ = 0.999.

#### 4.1.2. Data sets

The proposed method is evaluated on two datasets of CelebA (Liu et al., 2015) and CelebA-HQ (Karras et al., 2017). The CelebA (Liu et al., 2015) face dataset is an open dataset from the Chinese University of Hong Kong, which contains 202,599 facial images of 10,177 celebrity identities, and all of them are well-labeled. It is a very useful dataset for face-related training. We randomly selected 40,000 of these faces for our experiment. The 40,000 images are divided into a training set of 36,000 images and a test set of 4,000 images. The CelebA-HQ (Karras et al., 2017) dataset is a high-quality version of CelebA. It is a celebrity face attribute dataset containing 30,000 face images. We randomly select 27,000 images as the training sample and 3,000 images as the testing sample.

To train our network, we used irregular masks based on the quick draw irregular mask data set (QD-IMD) (Iskakov, 2021). Moreover, when testing the network, the irregular mask data provided by Liu et al. (2018) was used to assess our training result. Note that the irregular mask set includes 12,000 masks, which were divided into six categories with different coverage rates, i.e., (0.01, 0.1],(0.1, 0.2],(0.2, 0.3],(0.3, 0.4],(0.4, 0.5](0.5, 0.6].

### 4.2. Performance comparison

To show the inpainting performance of the proposed method, we first introduced our evaluation indicators and then compared quantitative measurements, visual comparisons, and subjective evaluations separately.

The following six mainstream image inpainting methods are used to compare with the proposed network: CA (Yu et al., 2018): A model trained in two stages of coarse and fine precision, which used a contextual attention mechanism in a fine precision network in the form of two codecs in series. GMCNN (Wang et al., 2018): A generative multicolumn neural network architecture in the form of three codecs in parallel. MEDFE (Liu et al., 2020): A mutual encoder–decoder CNN with feature equalizations for joint recovery of architecture and texture. RFR (Li et al., 2020): An advanced image inpainting method in feature space with recurrent feature reasoning and knowledge-continued attention. MADF (Zhu et al., 2021): A U-shaped framework with mask-aware dynamic filtering for image inpainting with a point-wise normalization. LG-net (Quan et al., 2022): A multilayer network architecture for image inpainting to combine networks with different receptive fields, considering the complexity of missing regions.

#### 4.2.1. Evaluation methods

To objectively evaluate the inpainting performance of different inpainting methods, the following objective indicators are used to evaluate the inpainting quality under the same experimental conditions:

*l*_1_ loss function (Gao and Fang, 2011): By calculating the sum of the absolute difference between the inpainting image and the original image, the similarity between the two images at the pixel level can be evaluated.


$$\begin{array}{l} l_{1}=\frac{1}{n}\overset{n}{\underset{i=1}{\sum }}|y_{i}-\hat{y_{i}}|. \end{array}$$

Peak signal-to-noise ratio (PSNR) (Hore and Ziou, 2010): It is defined by the maximum possible pixel value *Z* and mean square error (MSE) between images.


$$\begin{array}{l} \{\begin{array}{c} MSE=\frac{1}{n}\overset{n}{\underset{i=1}{\sum }}{\left(y_{i}-\hat{y_{i}}\right)}^{2}\\ PSNR=10\log_{10}\left(\frac{Z^{2}}{MSE}\right) \end{array}, \end{array}$$

where *Z* is equal to 255. The value of PSNR is usually between 20 and 40. The higher the value, the better the quality.

Structural similarity (SSIM) (Wang et al., 2004): This index compares the SSIM between images based on a comparison of the brightness and contrast characteristics of the images, and it can be shown by:


$$\begin{array}{l} SSIM\left(y_{i},\hat{y_{i}}\right)=\frac{\left(2\mu_{y_{i}}\mu_{\hat{y_{i}}}+C_{1}\right)\left(\sigma_{{y_{i}}_{\hat{y_{i}}}}+C_{2}\right)}{\left({\mu_{y_{i}}}^{2}+{\mu_{\hat{y_{i}}}}^{2}+C_{1}\right)\left({\sigma_{y_{i}}}^{2}+{\sigma_{\hat{y_{i}}}}^{2}+C_{2}\right)}, \end{array}$$

where μ and σ represent the mean and variance of image pixels, respectively.

Frechet inception distance (FID) (Heusel et al., 2017): It is a performance index for calculating the distance between a real image and a modified image feature vector. The lower the FID score, the better the image quality generated, and the higher the similarity to the original image.

#### 4.2.2. Quantitative comparison results

For quantitative evaluation, *l*_1_ loss function (Gao and Fang, 2011), PSNR (Hore and Ziou, 2010), SSIM (Wang et al., 2004), and FID (Heusel et al., 2017) are evaluation metrics. The results are shown in Tables 1, 2.

**Table 1:** Quantitative comparisons of ours with the other six methods in CelebA.

	**Masks**	**CA**	**GMCNN**	**MEDFE**	**RFR**	**LG-net**	**Ours**
ℓ1(%)^†^	1–10%	1.77	1.54	1.43	1.57	0.44	0.39
	20–30%	5.28	3.01	3.72	3.74	2.45	2.19
	40–50%	7.92	4.63	7.64	6.51	5.31	5.11
	AVG	4.99	3.06	4.26	3.94	2.73	2.56
PSNR^‡^	1–10%	33.12	36.29	36.21	37.26	40.72	42.25
	20–30%	24.07	28.33	27.85	29.14	30.67	31.66
	40–50%	21.11	26.08	23.50	25.23	26.09	26.56
	AVG	26.10	30.23	29.19	30.54	32.49	33.49
SSIM^‡^	1–10%	0.971	0.977	0.990	0.990	0.995	0.996
	20–30%	0.901	0.928	0.945	0.952	0.962	0.986
	40–50%	0.853	0.895	0.844	0.899	0.911	0.913
	AVG	0.908	0.933	0.926	0.947	0.956	0.965
FID^†^	1–10%	2.14	0.82	0.79	0.85	0.40	0.41
	20–30%	6.82	2.26	3.21	2.73	2.11	2.07
	40–50%	12.39	4.51	7.19	5.22	4.60	5.02
	AVG	7.11	2.53	3.73	2.93	2.37	2.50

‡ Higher is better.

† Lower is better.

**Table 2:** Quantitative comparisons of ours with the other six methods in CelebA-HQ.

	**Masks**	**CA**	**GMCNN**	**MEDFE**	**RFR**	**LG-net**	**Ours**
ℓ1(%)^†^	1–10%	1.86	1.14	1.02	1.59	0.46	0.39
	20–30%	5.33	3.05	3.68	3.58	2.38	2.11
	40–50%	7.84	4.51	7.65	6.44	5.27	5.03
	AVG	5.01	2.90	4.12	3.87	2.70	2.51
PSNR^‡^	1–10%	32.66	35.96	36.13	36.39	40.04	41.53
	20–30%	23.94	28.52	27.75	29.07	30.54	31.33
	40–50%	21.98	25.89	23.47	25.09	26.01	26.55
	AVG	26.19	30.12	29.12	30.18	32.19	33.14
SSIM^‡^	1–10%	0.971	0.984	0.990	0.991	0.995	0.997
	20–30%	0.903	0.933	0.943	0.957	0.968	0.987
	40–50%	0.853	0.897	0.865	0.902	0.917	0.921
	AVG	0.909	0.938	0.932	0.950	0.960	0.968
FID^†^	1–10%	2.06	0.85	0.84	0.86	0.39	0.37
	20–30%	6.97	2.24	3.17	2.67	2.08	2.11
	40–50%	12.42	4.56	7.12	5.21	4.61	4.47
	AVG	7.15	2.55	3.17	2.91	2.36	2.31

‡ Higher is better.

† Lower is better.

Tables 1, 2 compare the parameters of the seven methods used in the CelebA and CelebA-HQ data sets under four different indexes. The smaller the *l*_1_ and FID index values, the better the quality of figures, and the larger the PSNR and SSIM index values, the better the quality of figures. Through quantitative analysis, we can see that, under different coverage and indicators, our method generally outperforms the others. Only when the GMCNN image inpainting method deals with facial images with large area coverage (more than 40% coverage), some evaluation indexes are better than our method. A possible reason is that the jump connection between the residual blocks in our network pays too much attention to the shallow feature information of the image and neglects the processing of the global semantics. In addition, our performance on PSNR and SSIM assessment was significantly better than the other methods, showing that the facial image recovered by our method was of high quality and had a high SSIM with the original image.

#### 4.2.3. Visual comparison

**Figure 5:**
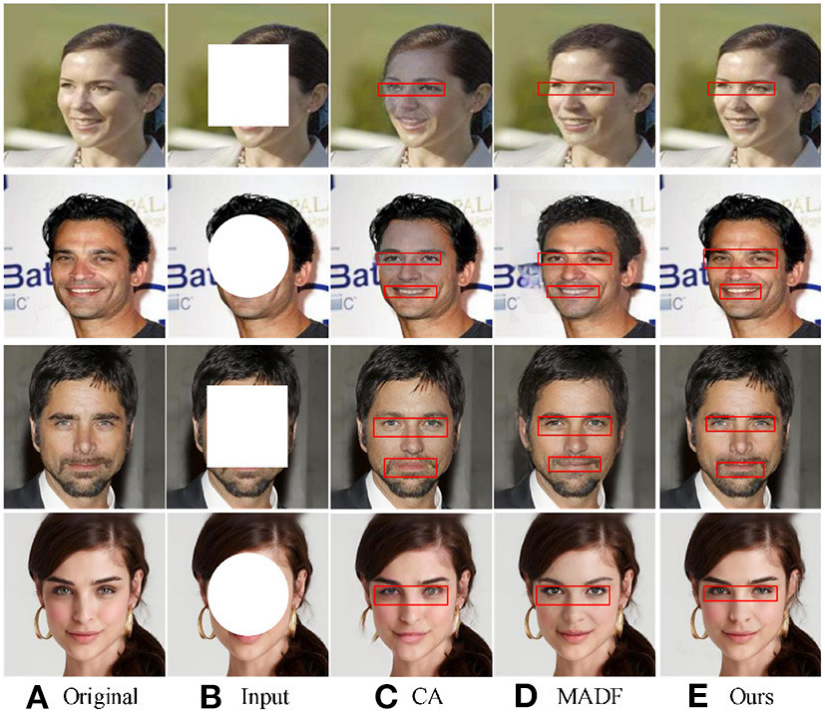
Visual comparison of different image inpainting methods on CelebA-HQ and ParisView datasets with regular masks. Obvious differences on the faces are highlighted by red boxes. The face images are adapted from the celeba-HQ dataset, which comes from Karras et al. (2017).

To better illustrate the inpainting effect, we compared the visual results from different image inpainting methods. As shown in Figure 5, the results of three different methods under the regular mask of CelebA are shown in the first and second lines, while the results of CelebA-HQ datasets are shown in the third and fourth lines. The hole size of the square mask was set as 128 × 128, and the radii of the circle mask was set as 64. From Figure 5, we found that facial images with rectangular masks restored by CA (Yu et al., 2018) and MADF (Zhu et al., 2021) tend to be fuzzy, and problems such as chromatic aberration and excessive discontinuity appear at the edges of the restored areas in the lips and eyeballs. However, facial images restored by the proposed method have clear facial features and good color consistency, making it difficult to distinguish the original image from the restored image with the naked eye. All these verify the effectiveness of the proposed method.

To further verify the inpainting effect of our method, we compared the inpainting performance of our method with other competitors on irregular masks.

**Figure 6:**
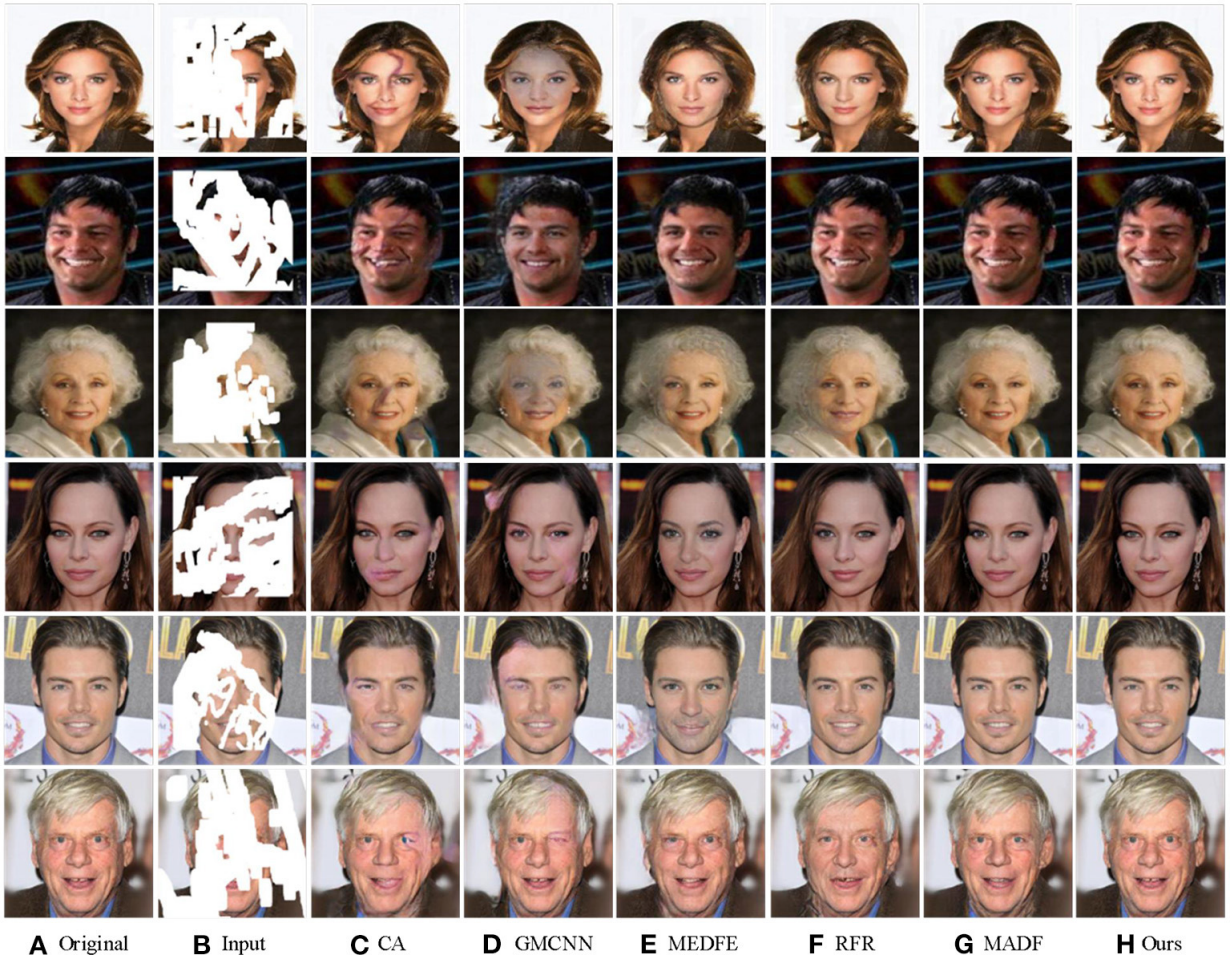
The comparison of different image inpainting methods on CelebA-HQ and ParisView with irregular masks. The face images are adapted from the celeba-HQ dataset, which comes from Karras et al. (2017).

The corresponding results are shown in Figure 6. The output images from the six different image inpainting methods of CelebA are shown in the first three lines, while the results from the CelebA-HQ dataset are shown in the last three lines.

Compared to the results generated with CA (Yu et al., 2018) and GMCNN (Wang et al., 2018), CLGN eliminated the phenomenon of blurring and distortion in the repair region, and the generation results was smoother and achieved a perfect transition from the damaged region to the undamaged region. MEDFE (Liu et al., 2020) and RFR (Li et al., 2020) offer excellent inpainting performance when the area to be repaired is small. However, for a large area of masks, they showed a wavy visual blur of water, which affects the overall observation effect of the inpainting image. Compared with MADF (Zhu et al., 2021), our GAM attention module-based method is more robust and stable depending on the good results of the local refinement network.

#### 4.2.4. User study

**Figure 7:**
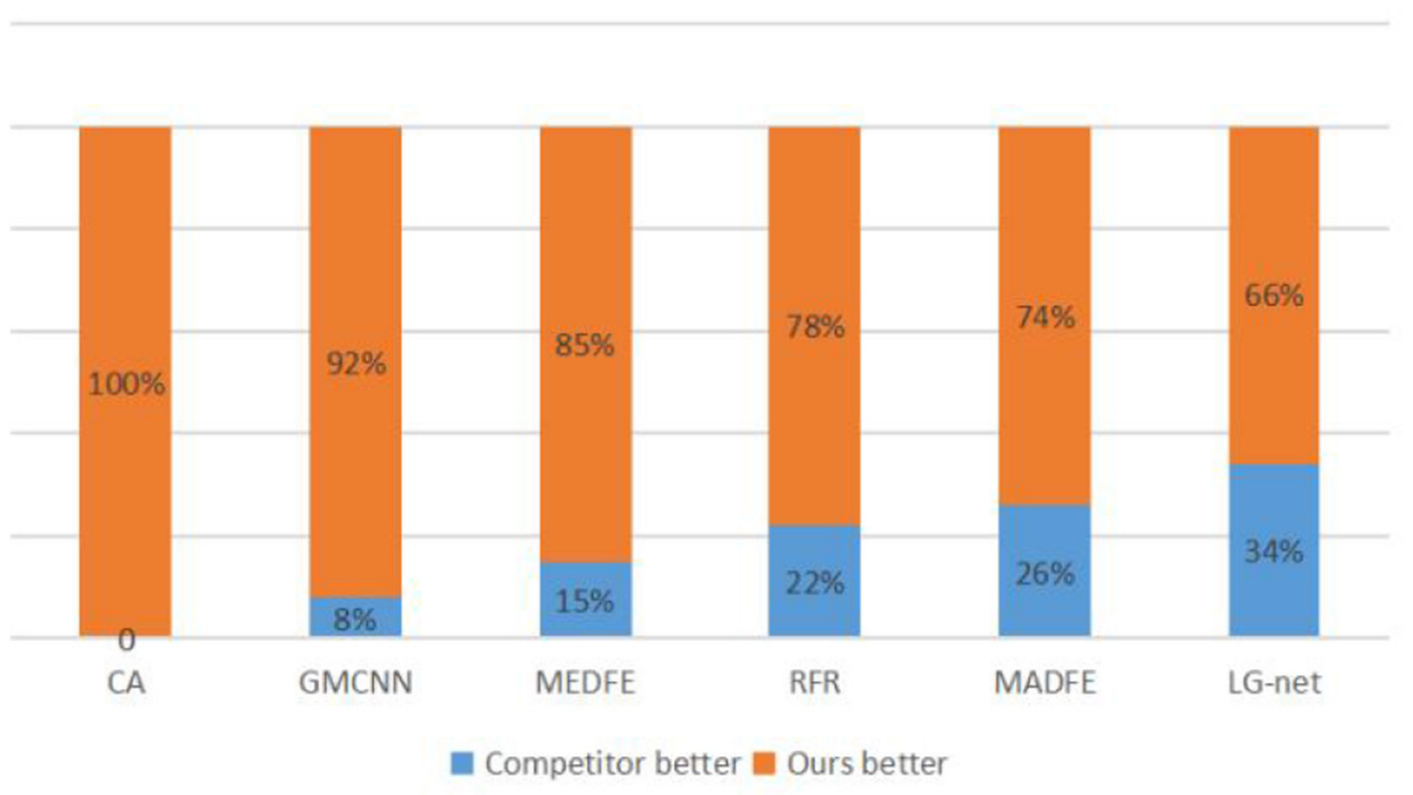
Statistical results from a user study on the CelebA data set. The value shows the percentage of each method chosen as the better one.

**Figure 8:**
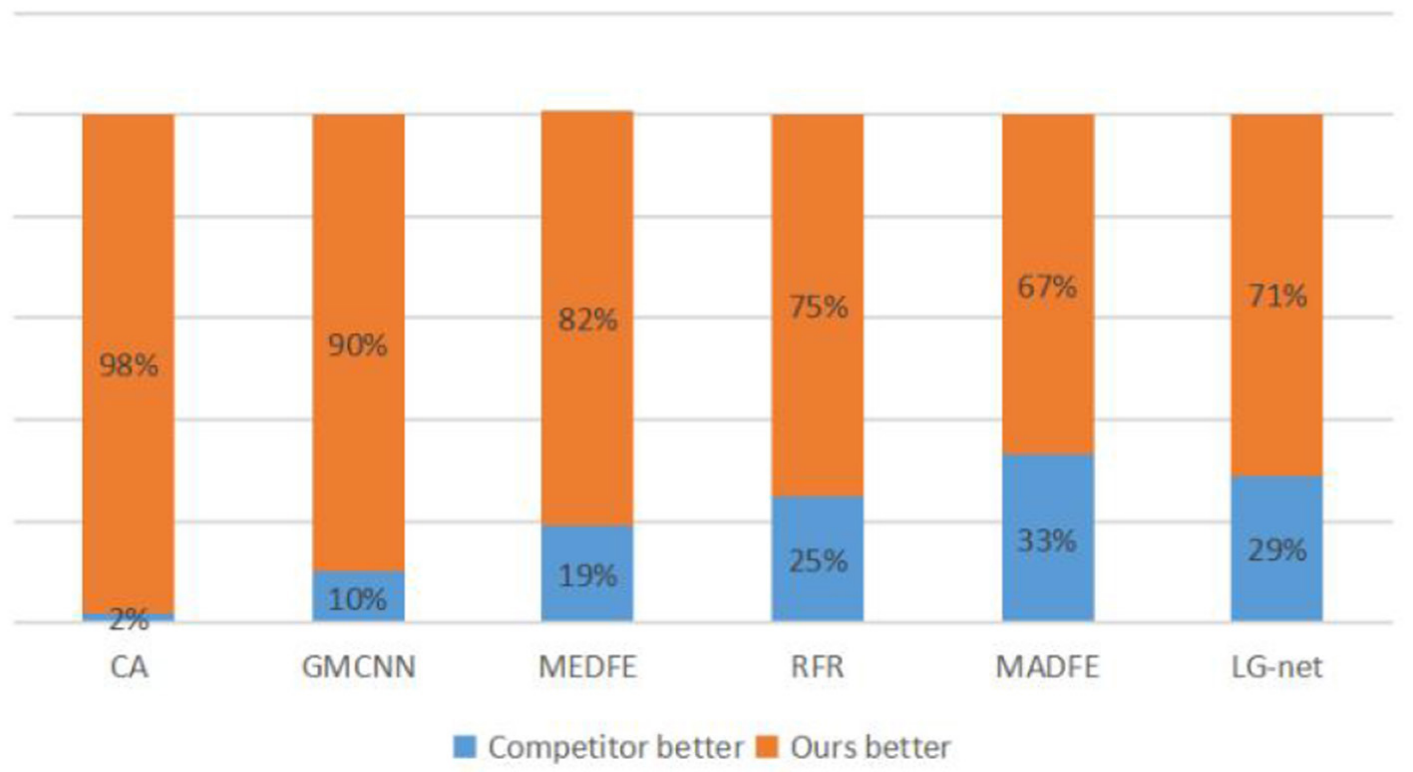
Statistical results of a user study on the CelebA-HQ data set. The value shows the percentage of each method chosen as the better one.

Because the evaluation metrics are not exactly fit human perception, we performed a user study on the Google Forms platform to further compare the visual quality of our method with six other mainstream image inpainting methods. For comparison, we randomly selected 10 pairs of CelebA (Liu et al., 2015) and CelebA-HQ (Karras et al., 2017), where each pair contains two inpainting images, one by a comparable method and another by our method. Note that the input images are covered by the same masked region. Then, we invited 24 volunteers for choosing the more natural and realistic images from each pair. In the end, we totally collected 2,880 votes. From Figures 7, 8, it can be concluded that our method is significantly more likely to be chosen than the other six methods, indicating that the visual quality of inpainting images of our method is superior.

### 4.3. Ablation studies

To verify the effectiveness of the loss function and a multistage network in our proposed method, ablation studies were performed on CelebA (Liu et al., 2015) and CelebA-HQ (Karras et al., 2017). The ablation experiment in this study as divided into three parts, which analyze the weighted loss network design, and GAM, respectively.

#### 4.3.1. Network design

**Figure 9:**
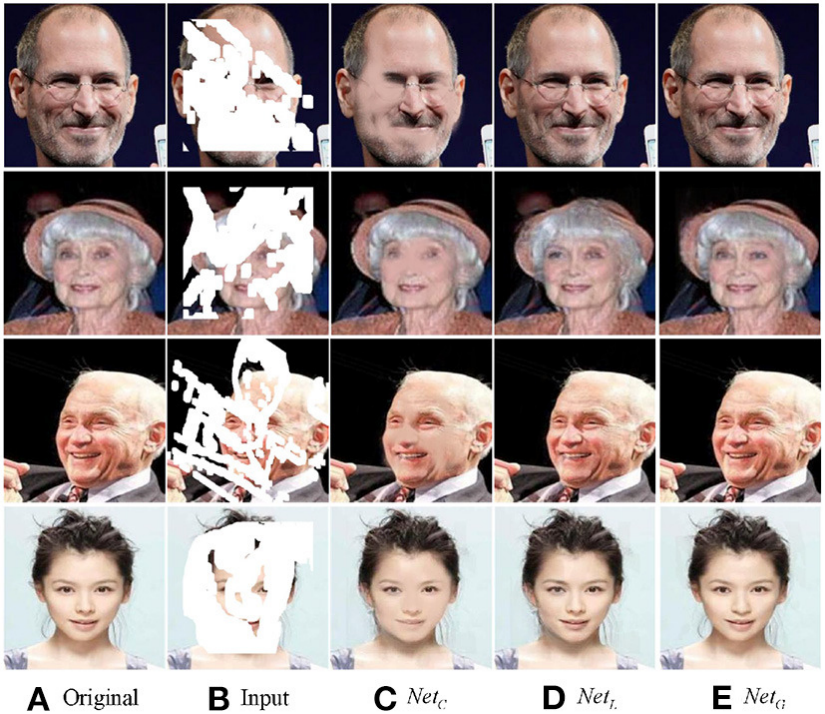
The output images of three subnetworks.

There are three subnetworks in our method: crude inpainting work *Net*_*C*_, local development network *Net*_*L*_, and a global attention-based network *Net*_*G*_. By comparing different variants of CLGN, the effectiveness of our network design can be verified and evaluated. Figure 9 shows the visual comparison, and Table 3 presents the corresponding numerical results. Note that we used incomplete images with one central square hole size of 128 × 128.

**Table 3:** The ablation of network design on CelebA and CelebA-HQ data sets.

**CelebA data set**					**CelebA-HQ data set**				
**Network**	**C**	**C** + **L**	**C** + **G**	**C** + **L** + **G**	**Network**	**C**	**C** + **L**	**C** + **G**	**C** + **L** + **G**
ℓ1(%)^†^	7.16	6.98	7.03	6.82	ℓ1(%)^†^	4.29	4.54	4.57	4.42
PSNR^‡^	23.01	23.05	23.08	23.14	PSNR^‡^	26.03	26.37	26.35	26.48
SSIM^‡^	0.948	0.971	0.969	0.980	SSIM^‡^	0.948	0.977	0.974	0.988

‡ Higher is better.

† Lower is better.

By comparing the results of “*Net*_*C*_” (*C*), “*Net*_*C*_+*Net*_*L*_” (*C* + *L*), “*Net*_*C*_+*Net*_*G*_” (*C* + *G*), “*Net*_*C*_+*Net*_*L*_+*Net*_*G*_” (*C* + *L* + *G*) in Table 4, we conclude that our proposed a multistage network, especially the global attention-based network, has a great effect on the inpainting results. This is probably because different types of networks can handle different types of visual artifacts. Hence, the more types and number of networks, the better the image processing effect.

**Table 4:** The ablation of attention mechanism on CelebA.

**Strategy**	**PSNR**	**SSIM**	**FID**	**LPIPS**
w/o GAM	21.43	0.892	11.28	0.203
w/one CBAM	21.68	0.907	7.90	0.169
w/one GAM	21.79	0.923	8.12	0.178
w/three CBAM	22.98	0.976	7.13	0.126

In addition, we analyzed our proposed method by comparing the inpainting results of three subnetworks and drew a conclusion from Figure 9 that the visual quality of the output images is getting better.

As shown in the first row of Figure 9, since the role of *Net*_*C*_ is to repair the image initially, the output image has a small range of blur. Moreover, *Net*_*L*_ removes local blur, especially those in the face by using the local information and *Net*_*G*_, finally, recovers complete semantic information and the image is restored to a maximum extent.

#### 4.3.2. Attention mechanism

To study the key role of GAM in the network, we conducted an ablation experiment on it. We attempted the following situations: remove the attention mechanism, place CBAM, and deploy GAM. The experimental results are presented in Table 4. From Table 4, it can be concluded that FID is greatly affected. while others are only a little affected by the attention mechanism. Moreover, compared to CBAM, GAM has an excellent effect on facial image inpainting.

**Figure 10:**
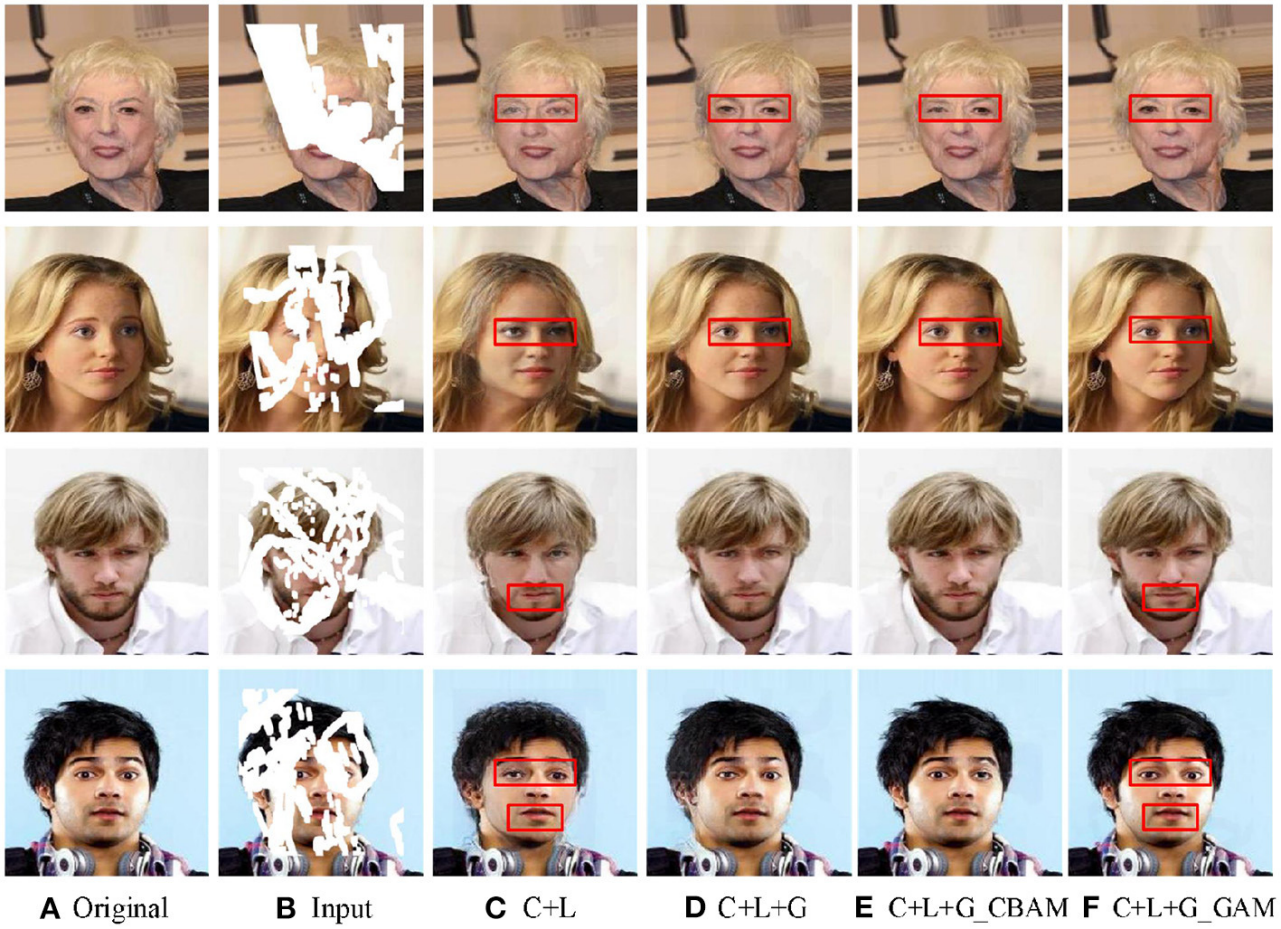
The outputs of different network frameworks with different attention mechanisms. Here “*C*” indicates a crude inpainting network, “*L*” means a local refinement network, and “*G*” means a global refinement network without any attention mechanism. “G_CBAM” means a global refinement network based on CBAM, while “G_GAM” means a GAM-based global refinement network. Obvious differences on the faces are highlighted by red boxes. The face images are adapted from the celeba-HQ dataset, which comes from Karras et al. (2017).

Next, we analyzed and compared the visual results of the different networks in Figure 10. From Figure 10, the results of “*Net*_*C*_+*Net*_*L*_” can only roughly repair the whole image, but there are artifacts or mismatches in the eyes, the mouth, and other parts. In contrast, attention mechanism-based network is more coordinated in global semantics and has a high similarity with the original image.

#### 4.3.3. Loss functions

In our study, we introduced five loss functions, namely reconstruction loss, adversarial loss, TV loss, perceptual loss, and style loss. Then, we conducted ablation experiments on the CelebA-HQ (Karras et al., 2017) dataset by removing these five loss functions from the network and analyzing the PSNR, SSIM, FID, and learned perceptual image patch similarity (LPIPS) values of the inpainting images. Note that we used incomplete images with one center square hole size of 128 × 128. From Table 5, it can be concluded that reconstruction loss plays the most critical role in performance optimization and that perceptual loss and style loss have the least impact on the performance of image inpainting.

**Table 5:** The ablation of loss functions on the CelebA data set.

**Strategy**	**PSNR**	**SSIM**	**FID**	**LPIPS**
w/o reconstruction loss	23.01	0.958	7.62	0.128
w/o adversarial loss	22.93	0.931	7.27	0.134
w/o TV loss	23.04	0.943	7.31	0.151
w/o perceptual loss	22.73	0.907	7.18	0.133
w/o style loss	22.98	0.972	7.23	0.136
All	23.14	0.980	7.11	0.124

## 5. Conclusions and future works

Facial image inpainting technology has practical significance in many fields. In this study, we proposed a multistage GAN (CLGN) for GAM-based facial image inpainting. This method combined the normalization of feature layer output in a deep convolutional GAN with the guidance of GAM to improve the robustness and accuracy of image detail recovery. As human faces have a common structure with different features such as the nose, the mouth, and the eyes, a multistage (crude-local-global) network can play the complete restoration role in distinct parts. Moreover, a skip connection was introduced using an encoder-decoder network to compensate for the loss of features due to down-sampling. The proposed method was compared with several inpainting methods on CelebA (Liu et al., 2015) and CelebA-HQ (Karras et al., 2017), and it had better performance than the mainstream traditional image inpainting method in both qualitative and quantitative analyses.

However, from the perspective of inpainting results, although our methods can predict a reasonable result according to the incomplete image, there are still some inevitable differences in color and texture details compared to the actual values. The guidance of structural information ensures its overall structure to some extent, but it is difficult to approach the true value for high-level semantic repairs such as the human eyes and mouths. From the perspective of the training process, large datasets can ensure that network training fits the model better, but at the same time, the long training time of the network becomes a thorny problem. Therefore, in subsequent study, we should focus on the facial image inpainting of higher semantics, which can ensure the credibility of the results and bring them closer to their actual value. At the same time, when designing a network for large data sets, network performance should be guaranteed and network training time should be minimized.

## Data availability statement

The original contributions presented in the study are included in the article/supplementary material, further inquiries can be directed to the corresponding author.

## Author contributions

XL: conception and design of study and drafting the manuscript. RL: writing—reviewing and editing. WZ: data curation and revising the manuscript critically for important intellectual content. EM: analysis and/or interpretation of data and software. All authors contributed to the article and approved the submitted version.
